# Autophagy and inflammation

**DOI:** 10.1186/s40169-017-0154-5

**Published:** 2017-07-26

**Authors:** Mengjia Qian, Xiaocong Fang, Xiangdong Wang

**Affiliations:** Zhongshan Hospital Institute of Clinical Science, Shanghai Institute of Clinical Bioinformatics, Fudan University Medical School, Shanghai, China

**Keywords:** Autophagy, Inflammation, Inflammatory diseases

## Abstract

Autophagy is a homeostatic mechanism involved in the disposal of damaged organelles, denatured proteins as well as invaded pathogens through a lysosomal degradation pathway. Recently, increasing evidences have demonstrated its role in both innate and adaptive immunity, and thereby influence the pathogenesis of inflammatory diseases. The detection of autophagy machinery facilitated the measurement of autophagy during physiological and pathophysiological processes. Autophagy plays critical roles in inflammation through influencing the development, homeostasis and survival of inflammatory cells, including macrophages, neutrophils and lymphocytes; effecting the transcription, processing and secretion of a number of cytokines, as well as being regulated by cytokines. Recently, autophagy-dependent mechanisms have been studied in the pathogenesis of several inflammatory diseases, including infectious diseases, Crohn’s disease, cystic fibrosis, pulmonary hypertension, chronic obstructive pulmonary diseases and so on. These studies suggested that modulation of autophagy might lead to therapeutic interventions for diseases associated with inflammation. Here we highlight recent advances in investigating the roles of autophagy in inflammation as well as inflammatory diseases.

## Introduction

Autophagy is a cellular process for the disposal of damaged organelles, denatured proteins as well as invaded pathogens through a lysosomal degradation pathway [1]. It was demonstrated to be activated during starvation or other stress response, including hypoxia, reactive oxygen species, DNA damage, protein aggregates, damaged organelles or intracellular pathogens. Through autophagy, cells can eliminate damaged or harmful components, thus it allows cells to survive in response to multiple stressors [2]. Autophagy has been implicated in a number of fundamental biological processes, including aging, immunity, development, and differentiation [3].

Besides autophagy, the cellular response to stress involves numerous other pathways, of which, the most common and important is inflammation. Inflammation plays protective or destructive roles in the response to endogenous or exogenous irritation or injury. It can be provoked by physical, chemical and biologic agents, including mechanical trauma, exposure to excessive amounts of sunlight, x-rays and radioactive materials, corrosive chemicals, extremes of heat and cold, or by infectious agents such as bacteria, viruses, and other pathogenic microorganisms. The pathogenesis of inflammation includes hemodynamic changes, leukocytes exudation, release of chemical mediators and hormonal response [4].

There are increasing evidences suggesting that autophagy plays critical role in the development and pathogenesis of inflammation and immunity response [5]. The autophagy machinery interfaces not only with most cellular stress-response pathways, but also entails direct interaction between autophagy proteins and immune signaling molecules [6]. The relationship between autophagy and inflammation is complex, both inductive and suppressive.

In this review, we summarized recent studies in autophagy and inflammation, and discussed the functions of the autophagy pathway and the autophagy proteins in inflammation and inflammatory diseases.

## Autophagy biology

### Concept understanding

Autophagy is a general term for pathways by which cytoplasmic material, including soluble macromolecules and organelles, is delivered to lysosomes for degradation. There are at least three different types of autophagy, including macroautophagy, chaperone-mediated autophagy and microautophagy [2]. Autophagy not only enables the reuse of intracellular constituents and supplies an amino acid pool during periods of starvation and stress response, but also helps to eliminate old/damaged organelles and extracellular organisms, thus provides basic cellular homeostasis. In addition, it was reported to play important roles in multiple pathophysiological processes including development, aging, and degeneration. Aberrant regulation of autophagy may result many diseases such as cancer [7], neurodegenerative diseases [8], and myopathies [9]. Recently, autophagy was found to be involved in immunity [5]. It can act as a direct effector by eliminating invading pathogens, regulating innate pathogen recognition, contributing to antigen presentation via major histocompatibility complex (MHC) class II molecules, and controlling B- and T cell development.

### Molecular regulation

**Fig. 1:**
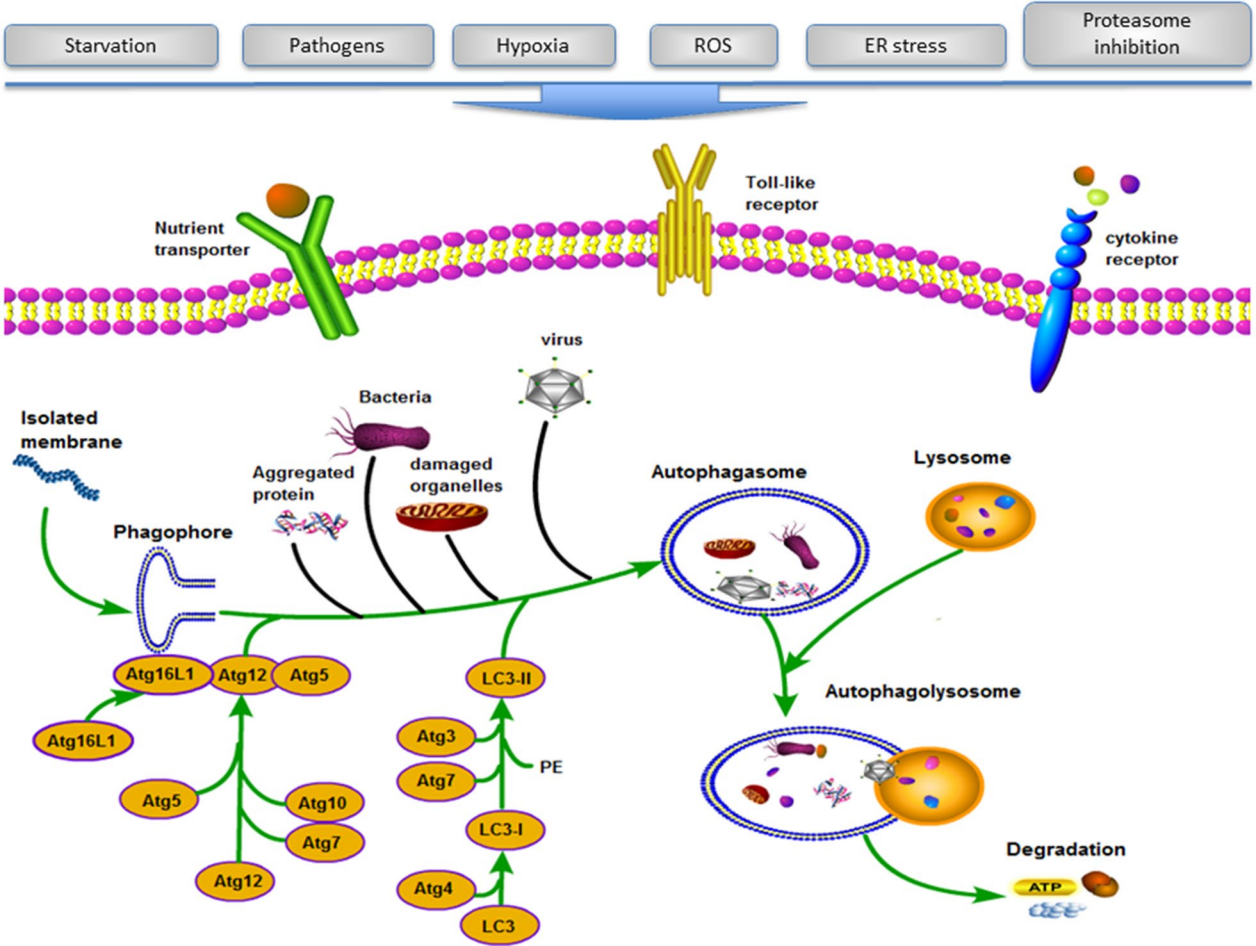
Induction and mechanisms of autophagy in mammalian cells. The process of autophagy involves two major steps: induction of autophagosome and fusion of autophagosome with lysosome. The ULK/Atg1 kinase complex, the autophagy-specific PI3-kinase complex, and PI(3)P effectors and their related proteins are important for the nucleation step, whereas the Atg12- and LC3/Atg8-conjugation systems are important for the elongation step. In addition, other proteins required for autophagosome-lysosomal fusion, lysosomal acidification, and lysosomal digestion, and regulatory signals that integrate environmental cues with the autophagic machinery are involved in autophagy

One breakthrough of the molecular mechanism for autophagy was achieved by identifying the genes in yeast, which are termed as autophagy-related genes (ATG) [10, 11]. These core Atg proteins are composed of four subgroups: (1) The Atg1/unc-51-like kinase (ULK) complex, which regulate the initiation of autophagy; (2) two ubiquitin-like protein [Atg12 and Atg8/microtubule-associated protein light chain 3 (LC3)] conjugation systems, which assist the elongation of the autophagic membrane; (3) the class III phosphatidylinositol 3-kinase (PI3K)/Vps34 complex I, which participate at the early stage of the autophagosome membrane formation; and (4) two transmembrane proteins, Atg9/mAtg9 (and associated proteins involved in its movement such as Atg18/WIPI-1) and VMP1, which may contributes to the delivery of membrane to the forming autophagosome [12]. The process of autophagy involves two major steps: induction of autophagosome and fusion of autophagosome with lysosome (Fig. 1). The ULK/Atg1 kinase complex, the autophagy-specific PI3-kinase complex, and PI(3)P effectors and their related proteins are important for the nucleation step, whereas the Atg12- and LC3/Atg8-conjugation systems are important for the elongation step.

### Measurement

Given this strong association between autophagy and different physiological and pathophysiological processes, there is a rapidly growing need among scientists to be able to accurately detect autophagy and to study its function. Details of the autophagy measurement methods have been reviewed elsewhere [13, 14]. For example, the number of autophagosome can be measured through electron microscopy, which is the most traditional and straightforward method [15]. However, the technique requires considerable specialized expertise since it is not easy to distinguish autolysosomes from endocytic compartments or from other vacuoles/structures once autophagosomes degradation processed. Immuno-gold labeling on ultrathin cryosections is a favorable approach to visualize autophagic structures, while specific antibodies that work properly with aldehyde fixation and the fragility of the autophagic structures are required, as well as the ultrathin cryosections techniques.

To fully understand a given biological process, it is usually critical to perform experiments to modulate the activity of the process. The autophagic activity can be inhibited or activated with agents that regulate autophagosome formation or subsequent degradation steps. However, right now we still lack highly specific autophagy inhibitors and activators. One of the most commonly used inhibitors is PI3-kinase inhibitors, including wortmannin, LY294002, or 3-methyladenine (3-MA). However, all of them are not autophagy specific and can meantime influence other cellular processes [16]. Another consideration for more specific inhibition of the autophagy pathway is to knockout or knockdown of different Atg genes, which has been reported in several studies [17, 18] and it is more specific than pharmacological agents. However even present at very low levels, some Atg proteins still function normally in autophagy, which may affect the experiment results and conclusions [19]. Thus, it is recommended that investigator not only confirm effective knockdown of autophagy protein expression levels with each siRNA, but also confirm effective inhibition of the autophagy pathway using a known autophagy-inducing stimulus such as starvation.

Given these potential limitations for each measurement, it is vital to state that none of these assays can be used alone to monitor or modulate cellular autophagic activity. In order to understand the effects of autophagy in a given biological settings, it is absolutely necessary to carry out multiple assays and compare the results of these investigations as a whole.

## Autophagy in inflammation

**Fig. 2:**
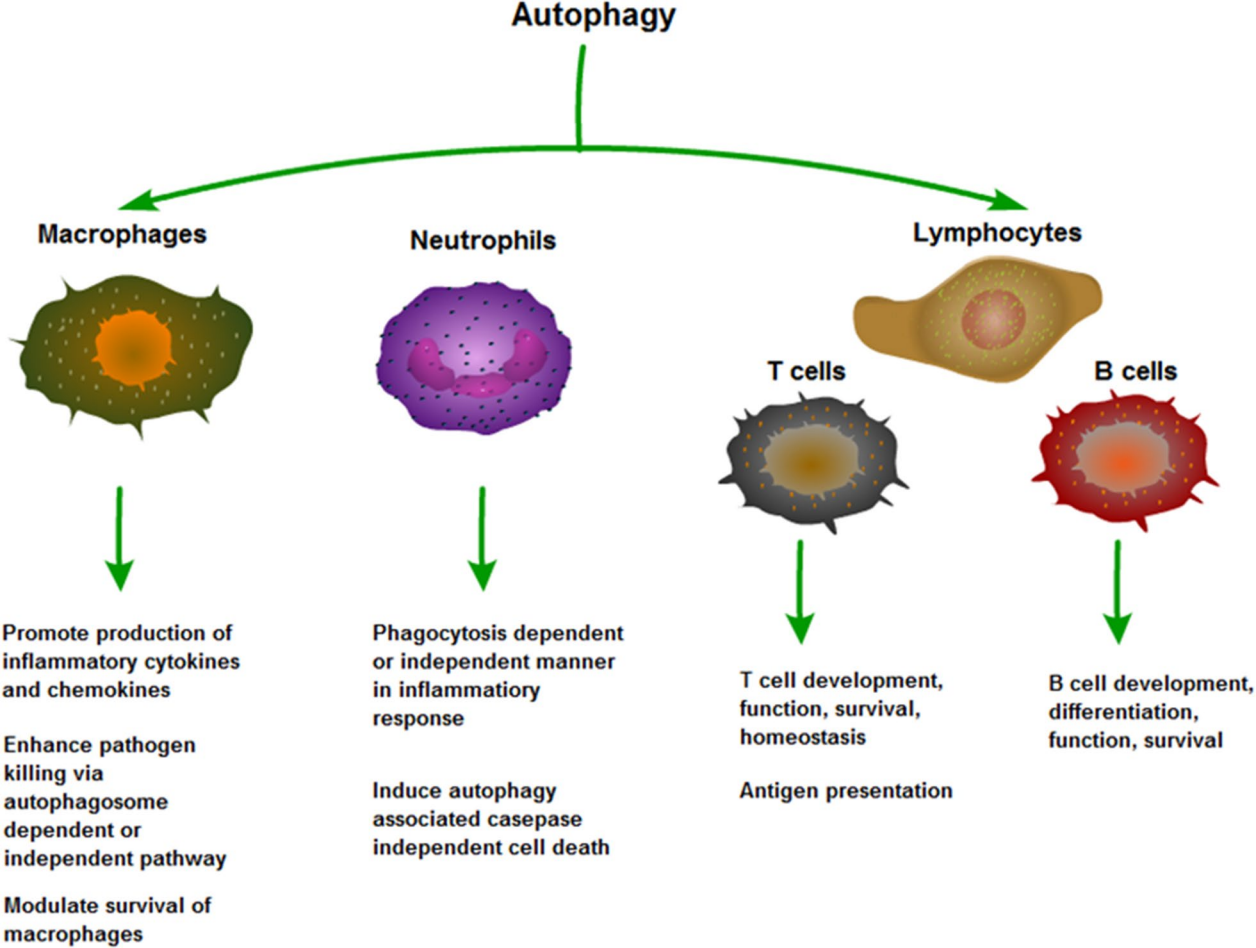
Effects of autophagy in inflammatory cells. Autophagy influences the development, homeostasis and survival of inflammatory cells, including macrophages, neutrophils and T lymphocytes and B lymphocytes

A complex association has been identified between autophagy and inflammation. First, autophagy influences the development, homeostasis and survival of inflammatory cells, including macrophages, neutrophils and lymphocytes, which play critical roles in the development and pathogenesis of inflammation (Fig. 2).

### Macrophages

Macrophage is essential for the host defense system. As a kind of phagocytes, it is able to uptake and kill pathogens intracellularly as well as producing inflammatory cytokine and chemokines [20]. Studies have shown that macrophages lacking Atg16L1 or Atg7, essential components of the autophagic machinery, appealed enhanced production of interleukin (IL)-1β and IL-18 in response to inflammatory stimulation through toll-like receptor (TLR) 3/4 signal pathway. Besides, the TLR signaling can also enhance phagosome maturation and the fusion of phagosomes and lysosomes depending on the autophagy pathway proteins ATG5 and ATG7, leading to rapid acidification and enhanced killing of the ingested organism in macrophages/monocytes [21, 22]. In mice, knockout of autophagy protein Atg5 in macrophages and neutrophils increases susceptibility to infection with *L. monocytogenes* and the protozoan *T. gondii*. Atg5 was required for interferon (IFN)-γ/LPS-induced damage to the *T. gondii* parasitophorous vacuole membrane thus killing intracellular pathogens via autophagosome-independent process [23]. Recent studies have shown that autophagy contributed to the execution of caspase-independent cell death in activated macrophages. The study detected an increase in poly (ADP-ribose) polymerase activation and reactive oxygen species (ROS) production in lipopolysaccharide +Z-VAD (a pan caspase inhibitor)—treated macrophages, followed by the formation of autophagic bodies and macrophage cell death. The death of activated macrophages could also be beneficial in controlling the level of inflammation [24].

### Neutrophils

Neutrophils are multifunctional cells, playing a central role in the innate immune system [25]. Inflammatory stimuli attract neutrophils to infected tissues where they engulf and inactivate microorganisms through the fusion of phagosomes with granules and the formation of phagolysosomes, in which antimicrobial peptides and ROS act synergistically for the clearance of pathogens [26]. In addition, neutrophil activation, degranulation and release of ROS into the extracellular medium, will lead to host tissue injury [27], while neutrophil apoptosis contributes to the resolution of inflammation [28]. There is evidence that autophagy occurs in neutrophils in both phagocytosis-independent and phagocytosis-dependent manner similar to that in macrophages [29]. However, the detailed mechanisms are not completely elucidated. So far, most of the studies focused on the role of autophagy in neutrophil death.

Recent studies have demonstrated that adhesion molecules induced autophagy-associated caspase-independent cell death in neutrophils, characterized by large cytoplasmic vacuolization and organelle fusion [30]. Such vacuolized neutrophils were observed in septic shock, cystic fibrosis, rheumatoid arthritis and several skin diseases [31], suggesting that induction of autophagy in these cells is a general phenomenon of neutrophilic inflammation response. Besides, neutrophil extracellular traps cell death (also named NETosis), is another type of programed cell death in neutrophils and involve NADPH oxidase activity. Recent studies have shown that inhibition of autophagy prevented NETosis via preventing intracellular chromatin decondensation, thus resulting in cell death characterized by hallmarks of apoptosis [32].

### Lymphocytes

Apart from innate immunity, autophagy also plays an indispensable role in adaptive immunity, including the development and homeostasis of the immune system and antigen presentation [33]. Several tissue-specific knockout models have been developed during the past few years to study the role of autophagy in T lymphocytes [34]. T cell receptor (TCR) activation is a strong trigger for autophagy in T lymphocytes. Meantime, autophagy-related genes are required for T cell proliferation upon TCR stimulation. T lymphocytes lacking Atg5, Atg7, Atg3 or Beclin-1 all showed impaired proliferation and enhanced cell death. The deficiency of Atg5 gene leads to the reduction of thymic cellularity and decreased number of peripheral T lymphocytes through enhancing cell death, suggesting the role of autophagic proteins in the regulation of T cell homeostasis [35]. Besides, autophagy also plays an important role in the selection and function of thymocytes. Studies have demonstrated that Atg5-deficient thymic epithelial cells underwent a disrupted process of positive and negative selection; moreover, when these cells transferred, they are apt to induce autoimmune diseases [36]. Via selectively degrading mitochondria [14] and endoplasmic reticulum [37], autophagy helps to maintain intracellular organelle homeostasis. Atg5-deficient T lymphocytes revealed a remarkable enrichment of the content of mitochondria, which was assumed to be the major reservoir of toxic reactive oxygen species [38]. Although it is demonstrated that autophagy is required for T cell survival, excessive autophagy seems to be destructive for T lymphocytes. Besides, autophagy is differentially regulated in each T helper subset. For example, Th2 cells are more resistant to growth factor-withdrawal cell death when autophagy is blocked [39]. Moreover, TCR-induced autophagy is compromised in aged CD4+ T lymphocytes while the mechanisms have been unclear [40].

Besides the indirect effects on the survival and function of T cells through autophagic proteins, autophagy also showed a direct role in antigen presentation to antigen-specific T cells (a process essential for the induction of acquired immunity) [41]. MHC class II molecules are localized on autophagosomes, and the autophagic machinery promotes the presentation of viral and self-antigens by MHC class II molecules to antigen-specific CD4+ T cells [42]. Upon infection by human simplex virus 1, autophagy controls the MHC class I-dependent presentation of viral antigens to CD8+ T cells [43].

Studies of autophagy in B lymphocytes are fewer than that in T lymphocytes. However, present studies on the role of autophagy in B lymphocytes have raised many interest and important questions for further investigation. As in T cells, Atg5 gene is also required in the development and survival of B lymphocytes. However, there is a study shown that Atg5 was only required for the maintenance of B-1a B cells, but not B-1b or B-2 B cells, and affected the number of pre-B but not pro-B cells [44], which suggested that Atg5 genes may play a critical role in the specific stages of B cell differentiation. Analysis of the expression of a Beclin 1-GFP transgene in T and B cells suggests that Beclin 1 may be involved in the development of lymphocytes and provides a critical link between apoptosis and autophagy. Beclin 1-chimeras had greatly reduced numbers of early B cells in the bone marrow compared with controls, while the peripheral B cell compartment was normal [45]. Recent studies indicated that autophagy was induced specifically by apoptotic B cell antigen receptor signaling [46].

## Autophagy and production of inflammatory mediators

### Regulation of autophagy by cytokines

Autophagic proteins have important roles in the regulation of inflammatory mediators and will affect cytokine production in macrophages [47]. In fact, it is well established that Th1 cytokines, including IFN-γ, TNF-α, IL-1, IL-2, IL-6 and TGF-β, have been shown to have the effects of autophagy inducement, while the classical Th2 cytokines, including IL-4, IL-10 and IL-13, have the effects of inhibition [48, 49]. Activation of macrophages with IFN-γ leads to the increased maturation of mycobacteria-containing phagosomes and autophagy in an Irgm1/IRGM-dependent manner [50], leading to increased intracellular killing of pathogens. However, IFN-γ-induced phagosome maturation can be abrogated by TNF blockers, which suggested that IFN-γ-induced phagosome maturation and autophagy might be TNF-α dependent. Interestingly, TNF-α is also demonstrated to play a role in stimulating autophagy in various cell types, while the actions and mechanisms are different between various cell types [51, 52]. For example, TNF-α can up-regulate the expression of the autophagy genes LC3 and Beclin 1 through activation of the Jun kinase signaling pathway as well as the inhibition of Akt activation [53]. TNF-α can also induct autophagy through ERK1/2 pathway [54, 55], while activation of NF-κB can inhibit TNF-α-induced autophagy, which is dependent on the generation of ROS [56]. On the contrary, studies have shown that the Th2 cytokines, like IL-4, IL-13 and IL-10, could inhibit starvation- or inflammatory stimulation-induced autophagy through different pathways. Inhibition of starvation-induced autophagy is dependent on the Akt pathway, while inhibition of IFN-γ or rapamycin-induced autophagy is dependent on STAT signaling pathway [57, 58]. In addition, other cytokines, chemokines and growth factors have also been implicated in the regulation of autophagy. TGF-β has been shown to induce autophagosome formation and can increase expression of autophagic mRNA, including Atg5, Atg7 [59]. However, the CC chemokine CCL2 (monocyte chemoattractant protein-1) and IL-6 both can stimulate autophagy and up-regulate anti-apoptotic proteins [60]. Moreover, IL-1 has also been demonstrated to stimulate autophagy [61]. However, insulin-like growth factor 1 [62] and fibroblast growth factor 2 [63] both can inhibit autophagy, while the detailed mechanisms still need to be further studied.

### Regulation of cytokines by autophagy

**Fig. 3:**
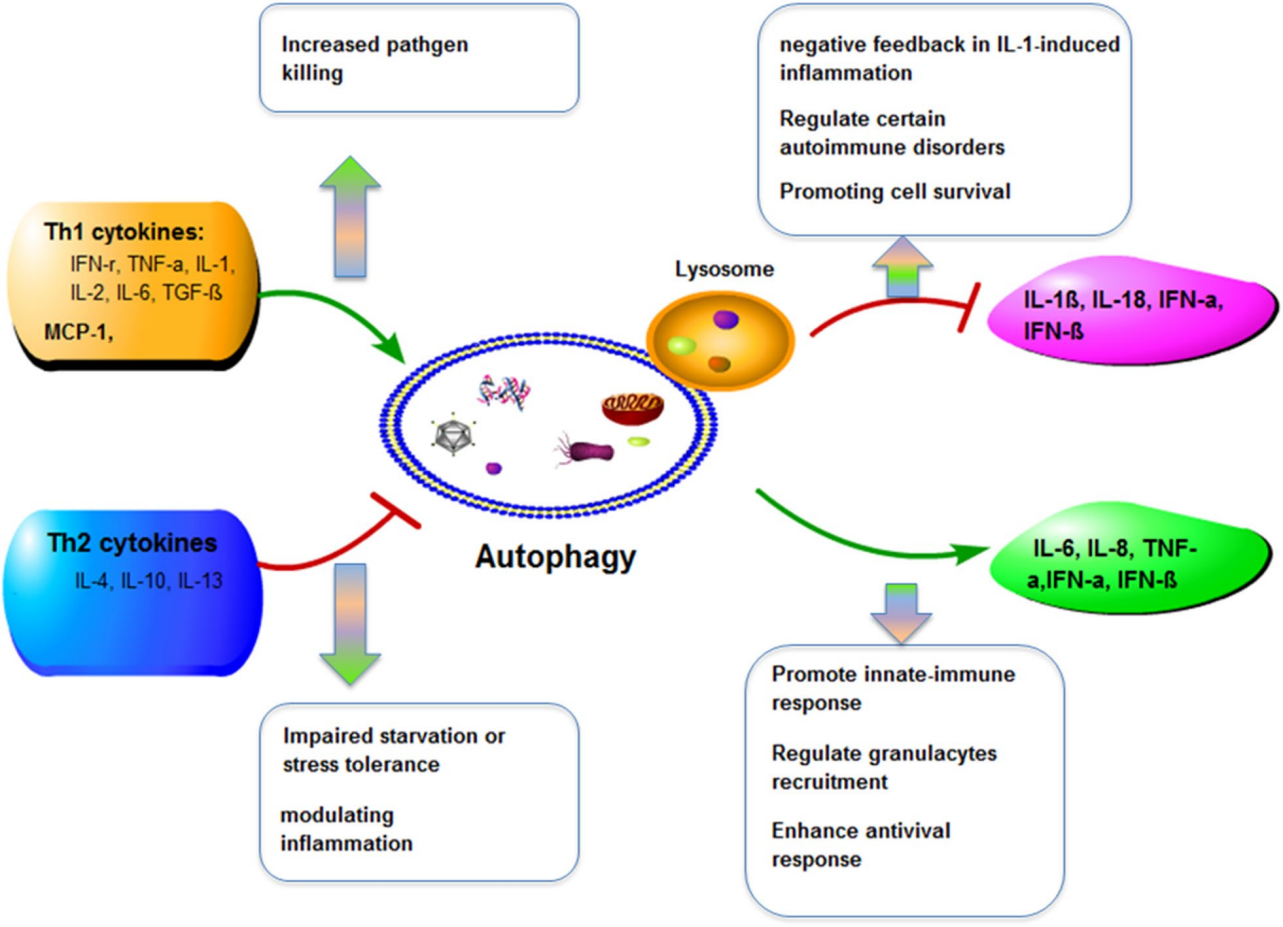
The interactions of autophagy and inflammatory cytokines or chemokines. Autophagy can affect the secretion of cytokines by itself, including Th1 cytokines, IFN-γ, TNF-α, IL-1, IL-2, IL-6, TGF-β, MCP-1 and Th2 cytokines, IL-4, IL-10 and IL-13, as well as other cytokines, IL-1β, IL-18, IFN-a, IFN-β, IL-8

Autophagy can affect the secretion of cytokines by itself (Fig. 3). Autophagy regulates IL-1β secretion through at least two separate mechanisms. Loss of autophagy in macrophages or dendritic cells, either through knock down of Atg7, Atg16L1 or Beclin 1, or by treatment with the autophagy inhibitor 3-MA, stimulates the processing and secretion of IL-1β in response to TLR agonists [64]. This effect may be dependent on TIR-domain-containing adaptor-inducing IFN-β (TRIF) and mitochondrial ROS and/or mitochondrial DNA and at least partially dependent on NLRP3 [65], and also may be independent of TRIF, but dependent on p38 MAPK signaling [66]. Conversely, induction of autophagy with rapamycin inhibits the secretion of IL-1β in murine dendritic cells in response to LPS with ATP or alum. Given that IL-1α and IL-1β have both been shown to induce autophagy, this may act as a negative feedback loop to control IL-1-induced inflammation. Similarly, the secretion of IL-18, IL-6 and TNF-α was also regulated by autophagy. Inhibition of autophagy enhanced the production of IL-18, but reduced the production of IL-6, IL-8 and TNF-α [67].

The modulation of autophagy in the secretion of IFN in virally-infected cells is controversial. Atg5 or autophagy deficient plasmacytoid dendritic cells was failed to produce IFN-α in response to infection with vesicular stomatitis virus (VSV) [68]. In contrast, other studies have demonstrated that embryonic fibroblasts from Atg5-/- mice are more resistant to VSV infection and produce higher levels of IFN-a and IFN-b mRNA in response to VSV or stimulation with dsRNA [poly(I:C)], compared with WT controls [69]. In hepatitis C virus infected hepatocytes, Atg7 knockdown induced IFN signal pathway, thus induced cell death [70, 71].

## Autophagy in acute and chronic inflammatory disease

Recently, emerging evidences have indicated that the process of autophagy may play an essential role in acute and chronic inflammatory processes, and thereby potentially impact the outcome of disease progression.

### Crohn’s disease

Crohn’s disease (CD) is a chronic and sometimes debilitating form of inflammatory bowel disease characterized by inflammation, ulceration, and neutrophil influx in the intestinal epithelia [72]. The underlying cause of CD is unknown; however it is clear that both environmental and genetic factors are required for its development. Recent studies have found links between autophagy related genes such as ATG16L, NOD2 and immunity-related p47 guanosine triphosphatase (IRGM) and the pathogenesis of CD through bioinformatics.

Nod2, a protein of the NLR family, functions as an intracellular bacteria sensor and was required in the induction of autophagy by bacterial peptidoglycan cell wall in intestinal epithelial cells [73]. Three major NOD2 variants are associated with CD; two missense mutations, R702W and G908R, and one frameshift mutation, L1007fsinsC. Human studies suggest that these NOD2 variants result in a loss of function [74]. A T300A variant in the ATG16lL gene, which plays a key role in autophagosome formation, has been identified as an associated risk factor for CD [75]. Another genome-wide association study suggested variants in the gene encoding IRGM were associated with CD [76]. Studies revealed variants of these genes may have been associated with the impaired clearance of harmful bacterial species associated with CD, impaired antigen presentation, and also with the higher production of proinflammatory cytokines implicated in the pathogenesis of CD, while further studies are still warranted to examine the contribution of these genes in the pathogenesis and treatment of CD.

### Infectious disease

Autophagy can exert anti-bacterial and anti-pathogen functions, which have already been demonstrated in several infectious diseases [77]. Take the case of *Mycobacterium tuberculosis* infection, the autophagy pathway and/or autophagy proteins have a crucial role in resistance to bacterial, viral and protozoan infection in metazoan organisms. *Mycobacterium tuberculosis* is an intracellular pathogen persisting within phagosomes through interference with phagolysosome biogenesis [78]; while experimental stimulation of autophagy can overcome the trafficking block imposed by *M. tuberculosis* [79]. Conversely, chemical inhibitors of autophagy will promote infection [80]. Additional studies have implicated the role of autophagy in defense against other microbial pathogens, such as *Legionella pneumophila* [81], *Dictyostelium discoideum* [82], *Shigella* [83] and so on.

### Pulmonary hypertension

Pulmonary arterial hypertension (PAH) is a complex disease of varying etiologies which characterized mainly by vasoconstriction, increased pulmonary artery pressure, thickening and fibrosis of the artery [84]. Recent studies have examined the prospective role of autophagic proteins in experimental models of PAH. Exposure to chronic hypoxia in mice resulted in the increased expression of LC3B and its conversion of LC3B-II in the lung. Increased LC3B staining was also observed in small pulmonary vessels of animals subjected to hypoxia. Moreover, hypoxic lungs contained elevated numbers of autophagosomes, as detected by electron microscopy. Importantly, mice genetically deleted for LC3B (LC3B-/-) displayed increased indices of pulmonary hypertension, including increased right ventricular systolic pressure, and Fulton’s index relative to wild-type mice, after chronic hypoxia [85]. LC3 exerts protective effects in the pathogenesis of PAH through hypoxia-specific inhibitory effects on the parameters involved in proliferative signaling (MAPK3/ERK1–MAPK1/ERK2 activation, VEGF secretion), as well as the inhibitory effects on pulmonary artery endothelial cells proliferation [86, 87].

### Cystic fibrosis

The pathological features of cystic fibrosis (CF) include aberrant accumulation of hyperviscous mucous in the airways, impaired mucociliary clearance, and increased inflammation partly due to the mutation of cystic fibrosis transmembrane conductance regulator (CFTR) [88]. Recent studies have demonstrated that human airway epithelial cells from CF patients, which bear the mutation in the CFTR gene, have an impaired autophagic response. Defective CFTR-induced upregulation of ROS and tissue transglutaminase drive the crosslinking of Beclin 1, leading to sequestration of PI3-K complex III and accumulation of p62 [89], which regulates aggresomal formation. Both CFTR knockdown and the overexpression of GFP-tagged-CFTRF508del induce Beclin 1 downregulation and defective autophagy in non-CF airway epithelia through the ROS-tissue transglutaminase pathway [90]. These data linked the CFTR defect to autophagy deficiency, leading to the accumulation of protein aggregates and to lung inflammation.

### Chronic obstructive pulmonary disease

Chronic obstructive pulmonary disease (COPD) is a chronic airway inflammatory disease characterized by progressive deterioration of lung function [91, 92]. More and more evidences have demonstrated that macroautophagy plays a significant and complex role in COPD pathogenesis [93, 94]. In lung biopsy specimens form patients with COPD, Western blot detected elevated level of LC3b-II protein when compared with non-COPD control patients. The level of LC3b-II correlated positively with clinical severity as measured by global initiative for COPD score [95]. Further studies confirmed that exposure of lung epithelial cell lines and fibroblasts to cigarette smoke extract induced the accumulation of autophagosomes on electron micrographs and enhanced levels of LC3b-II protein [96]; while genetic depletion of two macroautophagy pathway members, Beclin-1 and LC3b, reduced the rate of cell death in cigarette smoke extract-exposed cells [97]. Besides, studies also found that the macroautophagic flux in macrophages from COPD patients was greatly inhibited, which may contribute to the excessive inflammatory response in airway [98].

### Other systemic inflammatory diseases

Genome-wide association studies have linked several single nucleotide polymorphisms in Atg5 to systemic lupus erythematosus susceptibility [99]. Systemic lupus erythematosus is a multifactorial, heterogeneous disease characterized by autoimmune responses against self-antigens generated from dying cells. However, further studies are needed to determine the link between autophagy and systemic lupus erythematosus pathogenesis. Studies also suggested that defects in autophagy might contribute to inflammation-associated metabolic diseases such as diabetes and obesity via effecting on endoplasmic reticulum stress and insulin resistance [100].

## Therapeutic potential and future perspective

The present review summarized the previous studies which discussed the role of autophagic processes in the pathogenesis of inflammation, including elimination of pathogens, regulation of innate or adaptive immune response. Besides, we also referred to the potential therapeutic role of autophagy in some inflammatory diseases. Recently, increasing evidences also identified its role in carcinogenesis [101]. These observations collectively implicate that autophagy is an important modulator of disease pathogenesis. However, although progress has been made in elucidation the role of macroautophagy in inflammation, our understanding of the molecular mechanisms and pathways of autophagy and its relationship with inflammatory of inflammatory disease is still quite primitive.

As with any other core cellular processes, turning basic science knowledge about autophagy into therapies is difficult because of the interdependent nature of biochemical pathways. However, from a clinical perspective, the contributions of macroautophagy to the pathogenesis of inflammation and inflammatory diseases have potential therapeutic and diagnostic implications. From a therapeutic standpoint, the possibility that macroautophagy may play different physiological roles is depending on the cell type; as well as the fact that its different functions in different inflammatory conditions will lead to the result that when simply providing a chemical stimulator or inhibitor of macroautophagy to patients, they could have unpredictable consequences, such as improving symptoms or getting worse. From a diagnostic standpoint, the fact that macroautophagy marker proteins such as LC3b are increased before the onset of apoptosis suggests that they might prove useful as early biomarkers of some inflammatory disease. Future research will focus on the detailed mechanisms of autophagy pathways in specific diseases, as well as the interaction of autophagy with other pathophysiological processes thus determining whether the autophagic pathway can be manipulated for therapeutic gain in the treatment of inflammatory diseases and/or other diseases including cancer.

## Abbreviations

MHC: major histocompatibility complex
ATG: autophagy-related genes
ULK: unc-51-like kinase
LC3: microtubule-associated protein light chain 3
PI3 K: phosphatidylinositol 3-kinase
GFP: green fluorescent protein
RFP: red fluorescent protein
TLR: toll-like receptor
IL: interleukin
ROS: reactive oxygen species
TCR: T cell receptor
IFN: interferon
LPS: lipopolysaccoride
VSV: vesicular stomatitis virus
CD: Crohn’s disease
PAH: pulmonary arterial hypertension
CF: cystic fibrosis
CFTR: transmembrane conductance regulator
COPD: chronic obstructive pulmonary disease
